# Comparative study of photocatalytic activities of hydrothermally grown ZnO nanorod on Si(001) wafer and FTO glass substrates

**DOI:** 10.1186/s11671-015-1063-4

**Published:** 2015-09-16

**Authors:** Eun Hee Jeon, Sena Yang, Yeonwoo Kim, Namdong Kim, Hyun-Joon Shin, Jaeyoon Baik, Hyun Sung Kim, Hangil Lee

**Affiliations:** Department of Chemistry, Molecular-Level Interface Research Center, KAIST, Daejeon, 34141 Republic of Korea; Beamline Research Division, Pohang Accelerator Laboratory (PAL), Pohang, 37673 Republic of Korea; Center for Nanomaterials, Sogang University, Seoul, 04107 Republic of Korea; Department of Chemistry, Sookmyung Women’s University, Seoul, 04310 Republic of Korea

**Keywords:** ZnO nanorods, Photocatalytic oxidation, TEM, STXM, HRPES

## Abstract

ZnO nanorods have been grown on Si(001) wafer and fluorine-doped tin oxide (FTO) glass substrates for 1 and 4 h with the hydrothermal methods. The morphologies and photocatalytic activities of the ZnO nanorods were found to depend on the substrates. We investigated their properties by using spectroscopic analysis and demonstrated that the shape of nanorod and the ratios of external defects can be controlled by varying the substrates. Our experiments revealed that the nanorods grown on Si(001) have a single-crystalline wurtzite structure with (002) facets and that the number of surface oxygen defects increases with their length as the growth time increases. The nanorods grown on Si(001) have different facets, in particular wider (002) facets, and a higher ratio of the oxygen defect than the nanorods on FTO glass substrate. Moreover, the photocatalytic activities with respect to 2-aminothiophenol (2-ATP) of these nanorods were investigated with high-resolution photoemission spectroscopy (HRPES). We demonstrated that their photocatalytic activity is influenced by the ratios of surface oxygen defects, which varies with the substrate surface.

## Background

Zinc oxide (ZnO) has a direct wide band gap (3.37 eV) and a high electron hole binding energy (i.e., a high exciton binding energy) (60 meV) [1–3]. Recently, ZnO nanostructured materials have increasingly been investigated and have found various applications including UV nanolasers [4, 5], field emission devices [6, 7], dye-sensitized solar cells [8, 9], and photodetectors [10]. ZnO nanostructures have various shapes, such as nanowires, nanobelts, and nanorods [11–14].

Among these materials, one-dimensional zinc oxide (ZnO) nanorods are influenced by the synthetic methodologies involving chemical vapor deposition, the hydrothermal method, and vacuum plasma synthesis [15, 16]. The hydrothermal method can produce the nanorods with various shapes and orientations on the substrates ranging from well-aligned to random, while nanorods on the different substrates can be the same crystallinity. Moreover, their morphologies vary with the rate of facet growth, which depends on the concentration of oxygen defects involving oxygen vacancies, interstitials, impurities etc. [17–19]. Hence, the types of substrates and the types of crystal facets influence the catalytic activity of the synthesized nanorods. These nanorods have wide utilizations such as nanolasers, solar cells, optoelectronics, light-emitting devices, sensors, and photocatalysts [4–6, 8, 10, 11, 20], while bulk ZnO only absorbs in the UV region because of its large bandwidth, 3.2 eV (380 nm) [17–19]. Many researchers have attempted to enhance the photocatalytic activity of ZnO with respect to the concentration of oxygen defects, facets, and surface area depends on the substrates [18, 21]. Factors such as the ratios of exposed facets with surface oxygen defects (external oxygen vacancies) on ZnO are important for the photocatalytic activity. Therefore, a high ratio of surface oxygen defect (external oxygen vacancies) along with the length of the nanorods can result in higher photocatalytic activity [22, 23]. These factors can be regulated by the different substrate, typically, common non-metallic substrates that could be used in semiconductor such as sapphire and Si(001) wafer substrates were reported regarding ZnO [24, 25]. Si(001) wafer has been used as substrates for ZnO because of their low cost, although the lattice and symmetry matches between the Si(001) wafer substrate surface and ZnO are not as good as for sapphire [15, 26]. As a result, randomly oriented ZnO nanorods are grown on Si(001) wafer substrates, whereas sapphire substrates generate well-aligned ZnO nanorods with improved device performance [27]. In other words, improving the alignment of nanorods can improve the characteristics of the associated devices, and nanorod orientation is governed by the degree of lattice and symmetry matching between the substrate surface and ZnO [28].

In this paper, we investigated the photocatalytic activity by changing substrates of ZnO nanorods resulted in difference of the morphology, structure, and optical property. Therefore, we reported the preparation of ZnO nanorods with different orientations and morphologies on the Si(001) wafer and FTO (fluorine-doped tin oxide) glass substrates. We found that the variation in their morphology gives rise to significant variation in their photocatalytic activity with respect to the oxidation of 2-aminothiophenol (2-ATP). Here, we reported our analysis of surface oxygen vacancies (external defects) with high-resolution photoemission spectroscopy (HRPES) measurements in order to clarify the dependence of photocatalytic activity on morphology. HRPES techniques revealed the electronic structures of the interactions between 2-ATP and surface of the ZnO nanorods, and validated the exact reaction sites, which generated photocatalytic oxidation.

## Methods

### Preparation of ZnO nanorods

ZnO nanorod arrays were prepared on either fluorine-doped tin oxide (FTO) glass substrates or a Si(001) wafer substrates (phosphorus doped, 0.3~0.5 Ωcm). To increase the adhesion of the ZnO nanorods on the substrates, ZnO seed layers were coated onto the substrates before hydrothermal synthesis. Followed by heating on a hot plate at 100 °C for 5 min, 10 mM Zn(CH_3_COO)_2_∙2H_2_O (Junsei, 99%) solution in 1-propanol (Sigma-Aldrich, 99.7%) solution was spin-coated onto the substrates. Spin-coating and heating cycles were repeated by three times, and then the substrates were heated at 300 °C for 30 min. The seed layer coated substrates were kept in a Teflon-lined autoclave containing 25 mM Zn(NO_3_)∙6H_2_O (Junsei, 96 %), 25 mM hexamethylenetetramine (Sigma-Aldrich, 99 %), and 4 g/L polyethyleneimine (Sigma-Aldrich, branched, M_W_ ~25000) aqueous solution. The autoclave was placed in an oven at 90 °C for 1 or 4 h to perform the hydrothermal syntheses.

### Photocatalytic oxidation reactions

2-Aminothiophenol (C_4_H_4_SHNH_2_, Sigma Aldrich, 99 % purity) was purified by turbo pumping prior to dosing onto the three ZnO nanowrod samples. A direct doser controlled by means of a variable leak valve was used to dose the molecules and the same amount of oxygen molecule onto the ZnO nanorod arrays. The ZnO nanorod samples were irradiated with UV light (*λ* = 365 nm, VL-4.LC, Tube 1 × 4-Watt, Vilber Lourmat) through the quartz window of the vacuum chamber. The pressure of the chamber was maintained at 10^−6^ Torr during dosing, and the number of exposed molecules was defined by the dosing time in seconds: 1 L (Langmuir) corresponds to 1 s dosing under 10^−6^ Torr.

### Characterization

**Scheme 1:**
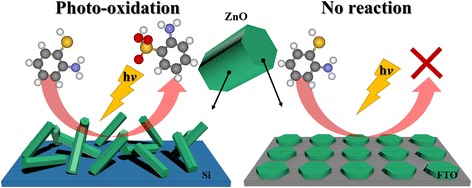
Schematic diagram of the oxidation reaction of 2-ATP using ZnO nanorods

The X-ray diffractions (XRD) of the ZnO nanorods were obtained with a Rigaku D/Max-A diffraction meter by Ni-filtered Cu K*α* radiation. The morphologies of the samples were characterized by performing field-emission scanning electron microscopy (FE-SEM, JEOL JSM-7600F) at an acceleration voltage of 15 kV and field-emission transmission electron microscopy (FE-TEM, JEOL JEM-2100F(HR)) at an accelerating voltage of 200 kV. Scanning transmission X-ray microscopy (STXM) was performed at the 10A beamline at the Pohang Accelerator Laboratory (PAL). A Fresnel zone plate (ZP) with an outermost zone width of 25 nm was used to focus the X-rays onto the ZnO nanorods on the transmission electron microscopy (TEM) grids. An order sorting aperture was installed between the ZP and the sample to collect the first order light diffracted by the ZP. The transmitted intensity was measured with a scintillation-photomultiplier tube (PMT). Image stacks were acquired at 1000–1060 eV and 520–570 eV with X-ray absorption spectroscopy (XAS) to extract the Zn *L*-edge and O *K*-edge spectra, respectively, which were obtained with exit slits of 20/20 and 20/15 μm (dispersive/non-dispersive), respectively. The STXM data were analyzed by using aXis2000 (open source software developed by the Hitchcock group, http://unicorn.mcmaster.ca/aXis2000.html). The Zn *L*-edge and O *K*-edge spectra of the nanorods were recorded for selected regions of interest in the image stacks to minimize the XAS contributions from the support films on the TEM grids. High-resolution photoemission spectroscopy (HRPES) experiments were performed at the 8A1 beamline at the Pohang Accelerator Laboratory (PAL), which was equipped with an electron analyzer (PHI-3057). The Zn 3*p*, S 2*p*, and O 1*s* core-level spectra were obtained by using photon energies of 160, 230, and 590 eV, respectively to enhance the surface sensitivity. The binding energies of the core-level spectra were determined with respect to the binding energy (*E*_B_ = 84.0 eV) of the clean Au 4*f* core-level for the same photon energy. The photoemission spectra were carefully analyzed by using a standard nonlinear least-squares fitting procedure with Voigt functions [29] (See this process, Scheme 1).

## Results and discussion

**Fig. 1:**
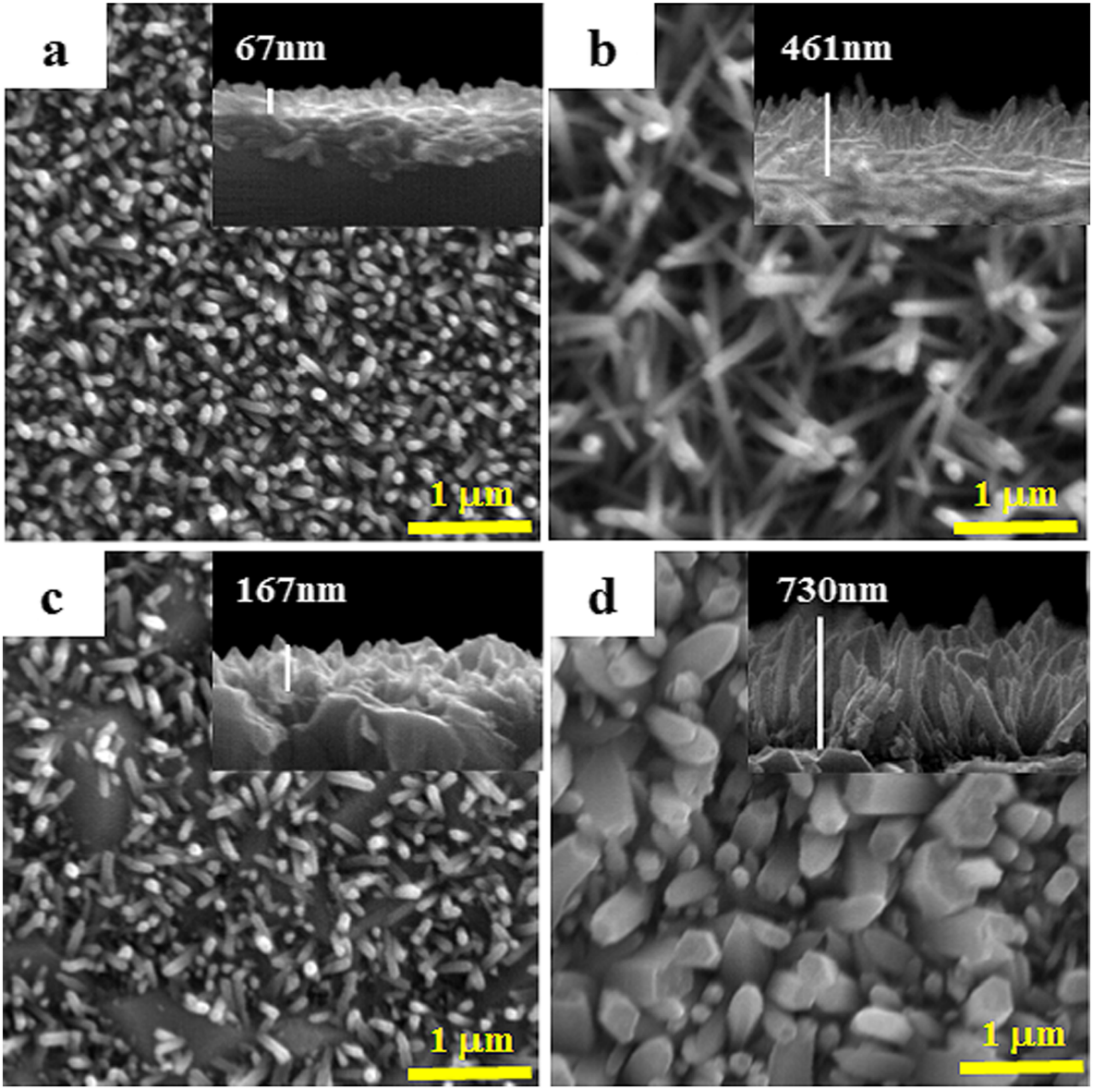
Cross-sectional and top-view SEM images of ZnO nanorod arrays grown on Si(001) wafer and FTO glass substrates: for 1 h, **a** ZnO-1/Si and **c** ZnO-1/FTO: for 4 h, **b** ZnO-4/Si and **d** ZnO-4/FTO

The SEM was used to examine the morphologies of the one-dimensional ZnO nanorods obtained with the growth times of 1 and 4 h on the Si(001) wafer and FTO glass substrates, respectively, and it was found that the grown nanorods cover the substrate surfaces (Now we denoted obtained ZnO nanorods with different growth times on different substrates as ZnO-1/Si, ZnO-4/Si, ZnO-1/FTO, and ZnO-4/FTO). It has been demonstrated that the ZnO nanorods exhibit randomly aligned growth on Si(001) wafer substrate due to the large lattice mismatch, whereas on FTO glass substrates ZnO nanorods have a slightly vertically alignment [4, 30–32]. The SEM image in Fig. 1a shows ZnO nanorods obtained after 1 h hydrothermal growth on a Si(001) wafer substrate. The cross-sectional SEM image in the inset shows that the nanorods on the Si(001) wafer substrate surface are 67 nm in height. When the growth time is increased from 1 to 4 h, ZnO nanorods longer than 67 nm are synthesized up to 461 nm, as shown in Fig. 1b. These ZnO nanorods have straight, needle-like shapes and are randomly aligned with respect to the substrate surface. For longer growth times, ZnO nanorods grow in a slightly vertically aligned array on the FTO glass substrate, as shown in Fig. 1d. The ZnO-4/FTO nanorods consist of one-dimensional nanostructures, 161 nm in length after 1 h of growth and 730 nm after 4 h of growth. Note that the ZnO-4/FTO nanorods have a wider diameter in the (100) facet with like the faceted hexagonal shape patterns than the ZnO-4/Si nanorods. Hence, these SEM images show that the structural morphologies of the ZnO nanorods on the Si(001) wafer substrates are distinctly different from that of the ZnO nanorods on FTO glass substrates. Moreover, as the growth time increases, the aspect ratio of each facet and the nanorod growth rate vary with the substrate. The SEM images imply that the substrate is one factor affecting the nanorod structural morphology and the defect ratio of each facet. Therefore, we will identify the photocatalytic activity over HRPES with 2-ATP molecule because we expect that ZnO-4/Si nanorods with a higher ratio of (002) facets exhibit a higher photocatalytic activity.

**Fig. 2:**
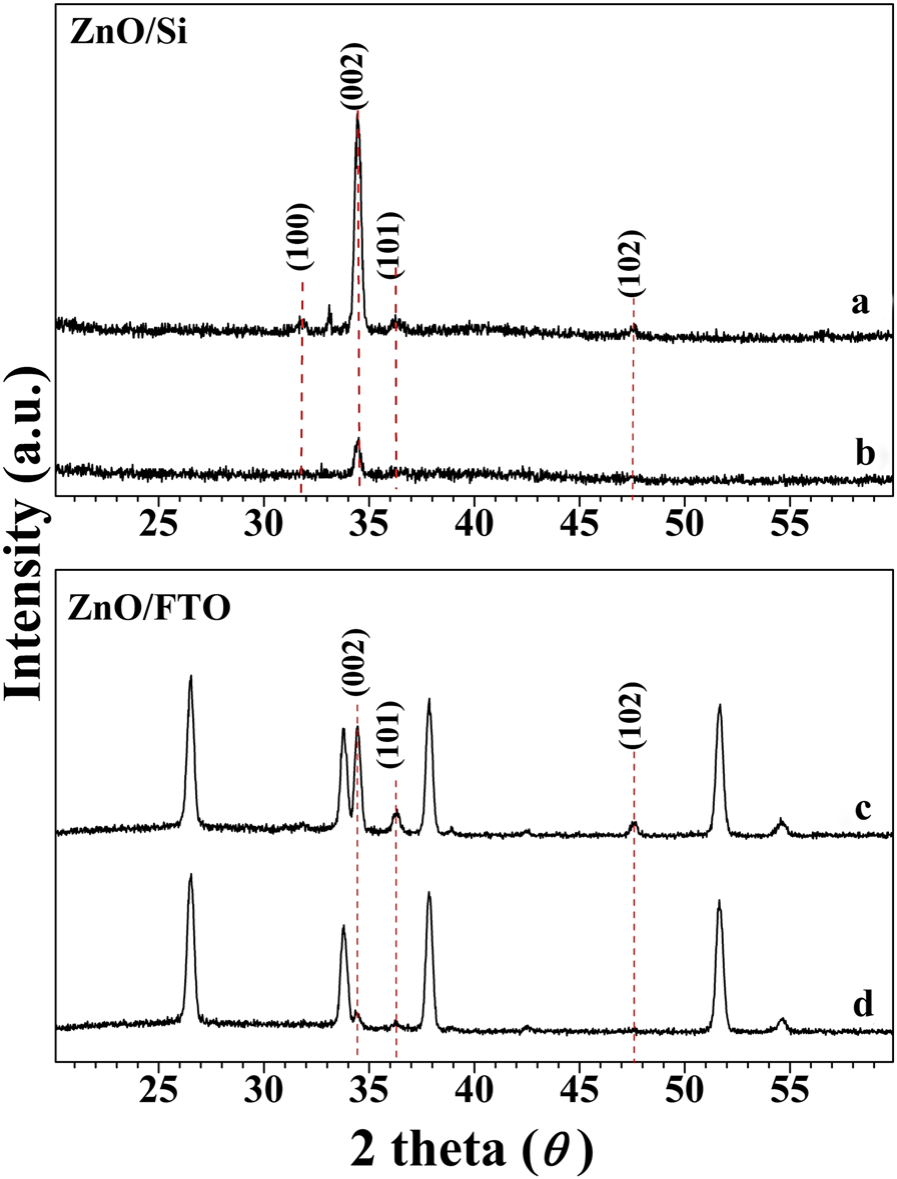
XRD patterns of ZnO nanorods: (*b* **)** and (*d*) grown for 1 h on Si(001) wafer and FTO glass substrates and (*a*) and (*c*) grown for 4 h on Si(001) wafer and FTO glass substrates

The crystal structures and (002) facet aspect ratios of the ZnO nanorods were characterized with X-ray diffraction (XRD). Figure 2 shows the XRD results for the ZnO nanorods grown on Si(001) wafer and FTO glass substrates. For both ZnO/Si and ZnO/FTO nanorods, four peaks arise at 2*θ* = 31.8°, 34.4°, 36.3°, and 47.6°, which are assigned to the (100), (002), (101), and (102) planes of wurtzite ZnO [33]. Moreover, the most intensive peak was observed at 2*θ* = 34.43°, which correspond to the (002) plane. The intensity of the (002) peak for ZnO nanorods grown for 4 h is very strong, more than five times higher than that of the ZnO nanorods grown for 1 h in each case. In Fig. 2, the relative intensity of the (002) peak for the ZnO-4/Si is stronger than that for the ZnO-4/FTO nanorods. Thus, we can expect that photocatalytic activity of the ZnO-4/Si nanorod is higher than that of the ZnO-4/FTO due to the ratio of the active site, (002) face. These results are in agreement with our SEM results. Hence, XRD results give a wurtzite ZnO pattern with an enhanced (002) peak corresponds to a high photocatalytic activity and the aspect ratio of the (002) facets is regulated by the substrate.

**Fig. 3:**
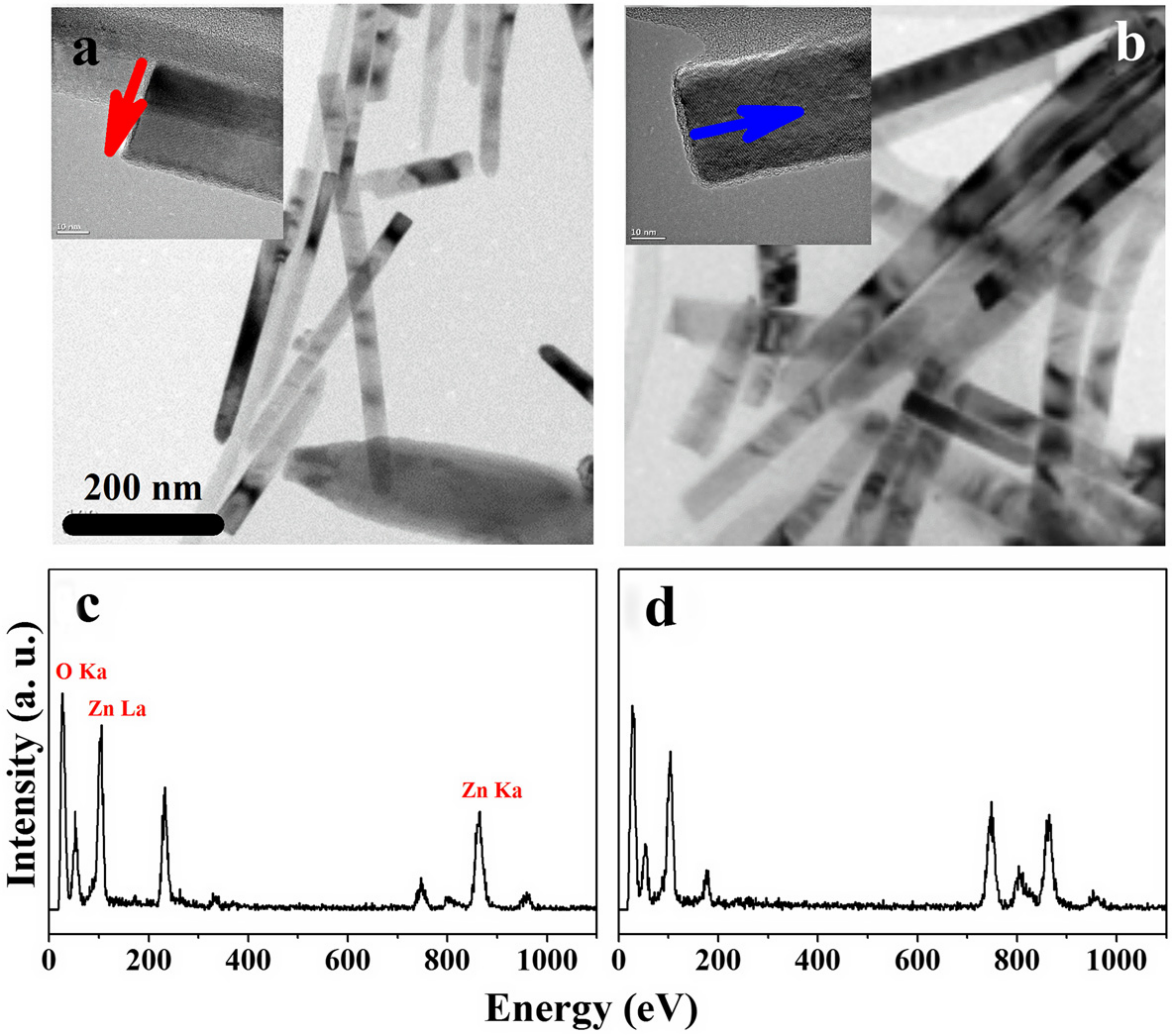
TEM images and the corresponding EDX spectra of ZnO nanorods grown for 4 h: **a** and **c** on the Si(001) wafer and **b** and **d** on the FTO glass substrate. We obtained these images and spectra of the nanorods after separating them from their respective substrates by using an ultrasonicator

We characterized the structures of single ZnO nanorods grown for 4 h on Si(001) wafer and FTO glass substrates by using TEM and energy dispersive X-ray spectroscopy (EDX) analysis. The EDX analysis confirms the presence of zinc and oxygen in the ZnO nanorods grown on the Si(001) wafer and FTO glass substrates. The morphologies of the ZnO nanorods grown for 4 h on the Si(001) wafer and FTO glass substrates can be clearly distinguished, as shown in Fig. 3 a, b. The TEM image of the ZnO-4/Si nanorods shows that the nanorods are straight with a single-crystalline structure like a short needle shape. The ZnO-4/FTO nanorods also have a slightly wider diameter in the (100) face (see the red arrow) and are longer in the [001] direction from the substrate (see the blue arrow) than the ZnO-4/Si nanorods. Moreover, the EDX ratios of oxygen and zinc are almost the same for the ZnO-4/Si and ZnO-4/FTO nanorods, as can be seen in Fig. 3c, d. Hence, although the ZnO-4/Si and ZnO-4/FTO nanorods have different morphologies, the total oxygen ratios are approximately the same. Thus, the nanorod morphology changes with the substrate; the substrate surface controls the aspect ratio of the (100) and (002) facets. The ZnO-4/Si nanorods have needle-like shapes, and the ZnO-4/FTO nanorods have a flat top shape due to the simultaneous growth of the both face (100) and (002), in Figs. 1 and 3. Thus, the crystallinity of the ZnO-4/FTO nanorods is lower than that of the ZnO-4/Si nanorods caused by the growth (100) face.

**Fig. 4:**
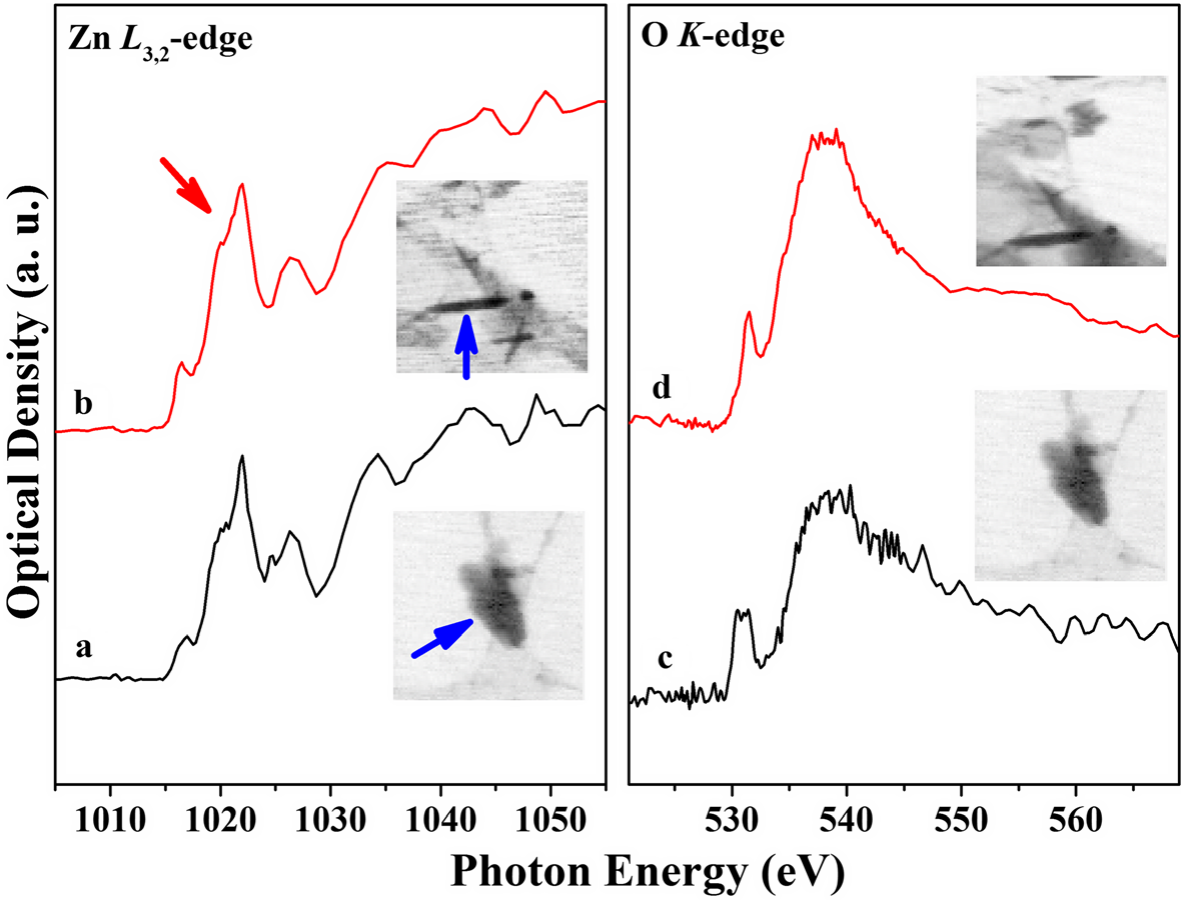
STXM images of the ZnO nanorods and their corresponding XAS spectra. The STXM images were obtained at 1021.3 eV (Zn L-edge) and 537.2 eV (O K-edge). XAS spectra: **a** Zn *L*_*3*_-edge and **c** O *K*-edge for ZnO-4/FTO and **b** Zn *L*_3_-edge and **d** O *K*-edge for ZnO-4/Si(001). We obtained these spectra of the nanorods after separating them from their respective substrates by using an ultrasonicator

To investigate the chemical compositions of the ZnO nanorods, we performed scanning transmission X-ray microscopy (STXM). Figure 4 shows the STXM images and the corresponding XAS spectra of ZnO-4/FTO and ZnO-4/Si to compare their local electronic structures and correlate them with the substrate surfaces. Figure 4 shows STXM images of the ZnO nanorods recorded at the Zn *L*_3_-edge (*E* = 1021.3 eV) and O *K*-edge (*E* = 537.2 eV) [34, 35]. The black regions (marked by blue arrows) in the STXM images in the insets of Fig. 4 originate from the ZnO nanorods at the fixed energies of the Zn *L*_*3*_-edge and O *K*-edge regions. Only the Zn *L*_3_-edge at the edge threshold (marked by red arrows) shows similarities; very strong Zn signals with similar features are evident for both ZnO-4/FTO and ZnO-4/Si nanorods. Hence, the Zn *L*_*3*_-edge can be clearly distinguished, and this part of the spectra near 1021.3 eV is due to transitions from Zn 2*p* to 4*s*/*d* [8]. Both the ZnO-4/FTO and ZnO-4/Si Zn *L*_*3*_-edge spectra indicate the presence of wurtzite ZnO structure in that the Zn *L*_*3*_-edge peak near 1021.3 eV is associated with strong Zn^2+^ signals in ZnO wurtzite structures [36, 37]. The local band structures of the ZnO nanorods are expected to be different from those of the bulk ZnO because of the size effect, structural distortion, and disorder, but we have confirmed that both ZnO-4/FTO and ZnO-4/Si have the same wurtzite ZnO nanostructures [38, 39].

The O *K*-edge XAS spectra of the ZnO-4/FTO and ZnO-4/Si nanorods are slightly different, whereas their Zn *L*_*3*_-edge XAS spectra have similar peaks. The intensity of the peak near 537.2 eV in the O *K*-edge spectrum of the ZnO-4/FTO nanorods is a slightly higher than that of the ZnO-4/Si nanorods and that of the ZnO-4/Si has slightly higher photon energy than the ZnO-4/FTO nanorods. The features of this spectrum are due to the transitions from O 1*s* to 2*p* [40]. In general, for excitation energies across the O *K*-edge, the band gap emission of the ZnO nanorods is enhanced while the defect emission increases at first (*E*_ex_ = 520–540 eV) and then decreases (*E*_ex_ = 540–575 eV) [41]. The defect emission is assigned to oxygen vacancies in the surface region of the ZnO nanostructures. The presence of this emission indicates that the excited state reached following O 1*s* to 2*p* excitation is coupled with oxygen defects [42]. Therefore, our XAS analysis shows the oxygen defect in the ZnO nanorod surface, and the ZnO-4/FTO nanorods contain almost the same ratios of the oxygen defects with regard to the ZnO-4/Si nanorods due to the similar intensity, as shown in Fig. 4c, d. Although there are slight differences between the O *K*-edge spectra of the ZnO-4/FTO and ZnO-4/Si nanorods, their O *K*-edge spectra demonstrate the presence of almost the same ratios of the oxygen defects. This XAS results are matched with XRD results.

**Fig. 5:**
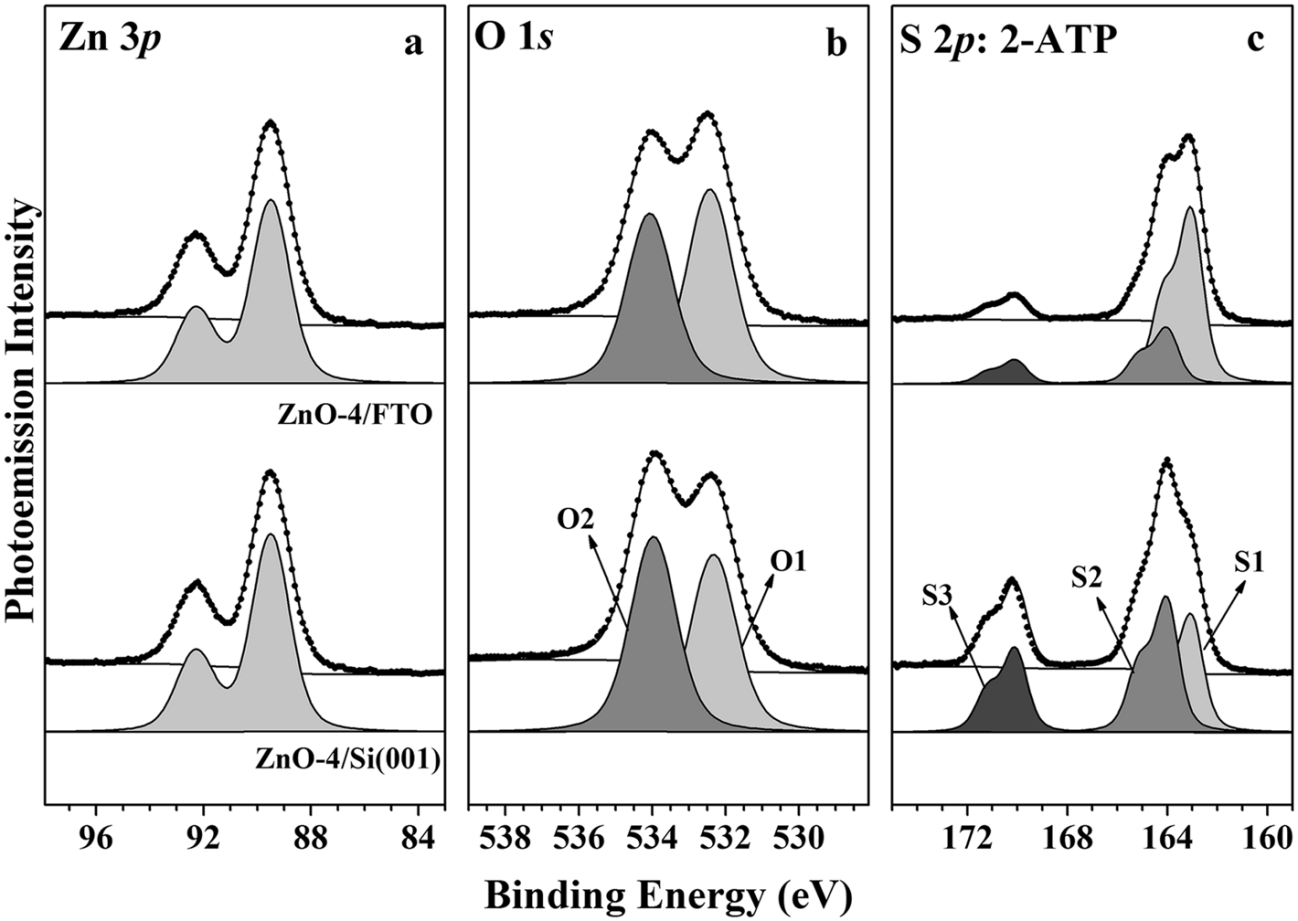
HRPES spectra: **a** Zn 3*p* and **b** O 1*s* core-level spectra for ZnO nanorods grown for 4 h on Si(001) wafer and FTO glass substrates, **c** S 2*p* core-level spectra for ZnO nanorod with adsorbed 2-aminothiophenol (2-ATP) of 360 L

The core-level spectra (Zn 3*p* and O 1*s*) of the ZnO nanorods were obtained with high-resolution photoemission spectroscopy (HRPES) to determine their electronic properties. Figure 5a shows the Zn 3*p* spectra for the ZnO nanorods on the Si(001) wafer and FTO glass substrates. The Zn 3*p* binding energy of the ZnO nanorods can be resolved into two peaks corresponding to Zn 3*p*_3/2_ (90.1 eV), Zn 3*p*_1/2_ (93.1 eV). [43] As shown in Fig. 5a, when the substrate surface is changed from the Si(001) wafer to FTO glass, there are no changes in the Zn 3*p* binding energy, and the original binding peaks in the Zn 3*p* core-level spectra appear in the same positions. Thus, there is no difference between the chemical state of Zn on the surfaces of the Si(001) wafer and FTO glass ZnO nanorods.

The O 1*s* spectra of the nanorods are shown in Fig. 5b. These spectra contain two distinctive features at 532 and 533.4 eV. These peaks are associated with oxygen defects (oxygen vacancies) (O1, 532 eV) and chemisorbed or dissociated oxygen species (O2, 533.4 eV), respectively [44–46]. The peak at 532 eV is related to the overall number of oxygen defects on both the (002) and (100) facets. According to the XPS results, the (O1) (532 eV) value of O 1*s* for ZnO-4/FTO is slightly higher than that of ZnO-4/Si nanorods. Moreover, the oxygen species giving rise to the peak at 533.4 eV (O2) are almost the same intensity due to the chemisorbed oxygen species between ZnO-4/Si and ZnO-4/FTO nanorods [8, 20]. Hence, the peak at 532 eV (O1) of O1*s* indicates the oxygen defects of the ZnO nanorods. It can be concluded that there are abundant surface oxygen defects in the ZnO nanorods. Although the intensity of oxygen defects in the ZnO-4/FTO nanorods is a slightly higher in the ZnO-4/Si nanorods, ZnO-4/Si with the high ratios of the (002) faces does exhibit effective photocatalytic activity, as shown in Fig. 5c.

We used HRPES to investigate the photocatalytic activities of the two types of ZnO nanorods with respect to 2-aminothiophenol (2-ATP), in particular to distinguish between nanorods grown on the Si(001) wafer and FTO glass substrates, which have different ratio of the (002) face, as shown in Fig. 2. The oxidation of the 2-ATP molecule was monitored by observing the changes in the S 2*p* peaks in the spectra, which were obtained at a photon energy of 230 eV. For 360 L of 2-ATP adsorbed on ZnO nanorods, there are three distinct S 2*p*_*3/2*_ peaks located at 163, 164, and 170 eV, which correspond to the thiol (C-SH) bounded peak (at 162.4 eV, marked S1), the thiol unbounded peak of 2-ATP (at 164.2 eV, marked S2), and sulfonic acid (SO_3_H) peak (at 170 eV, marked S3), which is related to the oxidation of 2-ATP, respectively [47–50]. As shown in Fig. 5c, when 2-ATP adsorbs onto the ZnO nanorods, there is a change in the S 2*p* peak intensity through the oxidation reaction. The intensity of the S3 peak at 170 eV increases more strongly for the ZnO-4/Si nanorods than for the ZnO-4/FTO nanorods. It was also found that as the intensity of the thiol (C-SH) peak decreases, a new peak corresponding to sulfonic acid (SO_3_H), which is the product of the oxidation of thiol, appears at 170 eV. The S3 intensity of the ZnO-4/FTO nanorods is lower than that of the ZnO-4/Si nanorods. Hence, the ratios of surface oxygen defects play a role in the photocatalytic activity of ZnO nanorods in the oxidation of 2-ATP.

The photocatalytic activity of the ZnO nanorods is regulated by the type of substrate; the Si(001) wafer substrate enables the growth of ZnO nanorods with a high ratio of (002) facets, whereas the FTO substrate produces nanorods with a wide (100) face compare with (002) face. Therefore, we demonstrated that ZnO-4/Si has a high ratio of the active site, (002) facets with oxygen defects, which can enhances the oxidation of the thiol (C-SH) group.

## Conclusions

In this study, we established a method for the synthesis of nanorods with different shapes on the Si(001) wafer and FTO glass substrates. Their nanorod crystal structures and morphologies were examined by using XRD, SEM, and TEM. The ZnO/Si nanorods have a wurtzite structure with a higher ratio of (002) facets than the ZnO/FTO nanorods due to the rod in shape with a wide (100) facet. Hence, the ZnO-4/Si nanorods have a higher ratio of surface defect with (002) facets than the ZnO-4/FTO nanorods. Their photocatalytic activities with respect to 2-ATP were examined with HRPES, and it was found that there are close correlations between the external oxygen defects of the ZnO nanorods, and differences between their substrate surfaces, and their photocatalytic efficiencies. We conclude that a high ratio of the defect with the (002) facet surface produce a substantial increase in the oxidation of 2-ATP. Hence, we confirmed that the differences between the photocatalytic activity of the ZnO/Si and that of the ZnO/FTO nanorods originate in their different ratios of the active sites, (002) facets with a high ratio of the oxygen defect. We demonstrated that the nanorods grown on the Si(001) wafer substrate surface have a high crystallinity, and that (002) facets with high ratios of the oxygen defects results in a high photocatalytic activity.

## Abbreviations

STXM: Scanning transmission X-ray microscopy
HRPES: High-resolution photoemission spectroscopy
